# Synergistic mechanism of GH11 xylanases with different action modes from *Aspergillus niger* An76

**DOI:** 10.1186/s13068-021-01967-1

**Published:** 2021-05-10

**Authors:** Shu Zhang, Sha Zhao, Weihao Shang, Zijuan Yan, Xiuyun Wu, Yingjie Li, Guanjun Chen, Xinli Liu, Lushan Wang

**Affiliations:** 1 State Key Laboratory of Microbial Technology, Institute of Microbial Technology, Shandong University, Qingdao, 266237 Shandong China; 2 State Key Laboratory of Biobased Material and Green Papermaking, Qilu University of Technology, Shandong Academy of Sciences, Jinan, 250353 Shandong China; 3 School of Life Sciences, Shandong University, Qingdao, 266237 Shandong China

**Keywords:** *Aspergillus niger*, GH11 xylanases, Transcription analysis, Degradation pattern, Synergistic hydrolysis

## Abstract

**Background:**

Xylan is the most abundant hemicellulose polysaccharide in nature, which can be converted into high value-added products. However, its recalcitrance to breakdown requires the synergistic action of multiple enzymes. *Aspergillus niger*, possessing numerous xylan degrading isozyme-encoding genes, are highly effective xylan degraders in xylan-rich habitats. Therefore, it is necessary to explore gene transcription, the mode of action and cooperation mechanism of different xylanase isozymes to further understand the efficient xylan-degradation by *A. niger*.

**Results:**

*Aspergillus niger* An76 encoded a comprehensive set of xylan-degrading enzymes, including five endo-xylanases (one GH10 and four GH11). Quantitative transcriptional analysis showed that three xylanase genes (*xynA*, *xynB* and *xynC*) were up-regulated by xylan substrates, and the order and amount of enzyme secretion differed. Specifically, GH11 xylanases XynA and XynB were initially secreted successively, followed by GH10 xylanase XynC. Biochemical analyses displayed that three GH11 xylanases (XynA, XynB and XynD) showed differences in catalytic performance and product profiles, possibly because of intricate hydrogen bonding between substrates and functional residues in the active site architectures impacted their binding capacity. Among these, XynB had the best performance in the degradation of xylan and XynE had no catalytic activity. Furthermore, XynA and XynB showed synergistic effects during xylan degradation.

**Conclusions:**

The sequential secretion and different action modes of GH11 xylanases were essential for the efficient xylan degradation by *A. niger* An76. The elucidation of the degradation mechanisms of these xylanase isozymes further improved our understanding of GH-encoding genes amplification in filamentous fungi and may guide the design of the optimal enzyme cocktails in industrial applications.

**Supplementary Information:**

The online version contains supplementary material available at 10.1186/s13068-021-01967-1.

## Background

Xylan is the most abundant hemicellulose polysaccharide in nature, accounting for about 20–35% of plant biomass [1]. It wraps around the outer layer of cellulose, together with lignin, resulting in “biomass recalcitrance” that makes plants resistant to microbial and enzymatic degradation [2–4]. Therefore, xylan must be degraded efficiently for the utilization of lignocellulose resources. The structure of xylan is composed of xylopyranosyl residues linked by β-d-1,4-glycosidic bond to form the main backbone, which is modified by various side-chain substituents such as l-arabinose, 4-*O*-methyl-glucuronic acid and acetyl group that are attached to the backbone in a variety of ways to form different bond types [1, 5]. The type and number of side-chain substituents vary in hardwoods (e.g., beechwood), herbaceous plants (e.g., wheat) and monocotyledons (e.g., corn) [6, 7]. Therefore, the synergistic degradation of heterogeneous xylan by a variety of backbone and side-chain degrading enzymes is an efficient strategy for converting it into soluble sugars.

Endo-β-1,4-xylanases are the most critical enzymes in the degradation of xylan backbone, breaking the β-1,4 glycosidic bond and producing different types of oligosaccharides [8], most of which belong to glycoside hydrolase (GH) 10 and 11 families. They differ greatly in their structures, substrate specificity and catalytic mechanism. GH10 xylanases adopt a (β/α)_8_-barrel fold, while the structures of GH11 xylanases are β-jelly roll [9, 10]. GH10 xylanases with relatively broad substrate specificity, are able to cleave the β-1,4 glycosidic bond closer to the side chain. GH11 xylanases catalyse xylan degradation specifically, which can better hydrolyze unsubstituted xylan regions [11, 12]. Moreover, GH11 xylanases can easily access complex biomass due to their low molecular weight and high catalytic activity [13]. These xylanases have been widely studied for their potential applications in various industries, including biofuel, textiles, pulp and paper technologies, food and medical industries [14–16]. In particular, xylanase hydrolysate xylooligosaccharides (XOS) can stimulate human intestinal health as a prebiotic [17, 18]. Most of the researches have focused on improving the thermostability of commercial enzymes so that they can adapt to harsh industrial environments [19, 20]. However, the catalytic activity and mode of action of these commercial xylanases are also closely related to industrial conversion and cost.

Filamentous fungi are potent producers of xylanases, with *Aspergillus* and *Thermomyces* being among the most prolific [21–23]. *Aspergillus* is one of the most important species in the degradation of hemicellulose, which generally contains several different xylanase genes in the genome and encodes multiple xylanases [24]. Recently, in *Aspergillus fumigatus*, a synergistic degradation mechanism of GH10 and GH11 xylanases has been proposed for the degradation of different parts of xylan [25]. In addition, the hydrolysates of GH11 xylanases can be further hydrolyzed by GH10 xylanases [26]. Gong et al. [27] have reported that *Aspergillus niger* An76 possesses a highly efficient xylan-utilizing system and in the process of degradation, the secretion of xylanases and side-chain degrading enzymes is in order and has a division of labor. Furthermore, it has a suite of five genes encoding xylanases, but proteomic analysis has shown that one GH10 and two GH11 xylanases are abundant during growth on xylan [27, 28]. However, an extensive biochemical and enzymatic characteristics study of each xylanase isozymes in the same microorganism is necessary to determine an optimal mixture of the enzymes with a suitable ratio for industrial application.

In the present study, a transcriptional analysis at the gene level and biochemical determination at the protein level was applied to explore multiple xylanases of *A. niger* An76 in xylan bioconversion. First, the transcription of various xylanase-encoding genes was analyzed by quantitative real-time PCR (qRT-PCR). Second, all five xylanases were heterologously expressed and biochemically characterized (except one GH11 xylanase with no activity) to explore the different catalytic performances. Moreover, structural bioinformatic analysis was carried out to distinguish the degradation patterns of three GH11 xylanases. These findings provide novel mechanistic insights into the synergistic degradation of xylan by *A. niger* An76 GH11 xylanases and facilitate the optimization of glycoside hydrolase cocktails for biomass conversion.

## Results

### Quantitative transcriptional analysis of five xylanase-encoding genes cultured with different substrates

**Fig. 1:**
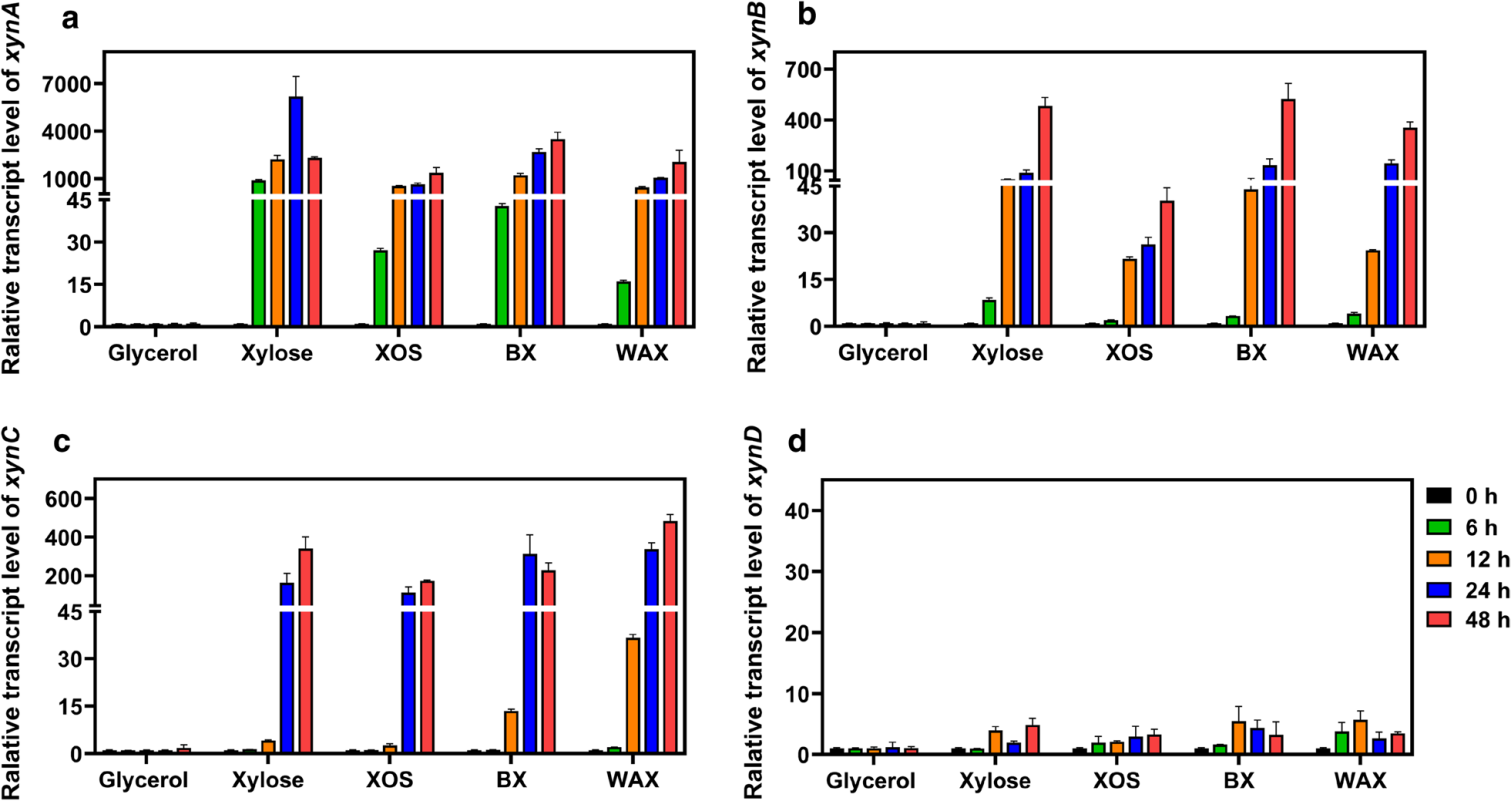
Relative transcript levels of four genes encoding xylanases in *A. niger* An76 induced by 1% glycerol, xylose, XOS, BX and WAX. The transcript levels of *xynA* (**a**), *xynB* (**b**), *xynC* (**c**) and *xynD* (**d**) in *A. niger* An76 at different time points (0, 6, 12, 24 and 48 h). Glycerol served as a control. Relative transcript levels were calculated by the 2^−ΔΔCT^ method. *T*-test analysis was used to calculate the significant differences in gene expression (*P* < 0.05)

According to genome analysis, *A. niger* An76 possesses one GH10 xylanase (XynC) and four GH11 xylanases (XynA, XynB, XynD, and XynE; Additional file 1: Table S1). All the five xylanases contain signal peptides, hence they are secretory proteins. To obtain further insight into the regulation of these xylanases expression and to complement the previous proteomic study [27, 28], we used qRT-PCR to determine changes in gene expression using glycerol, xylose, xylooligosaccharide (XOS), beechwood xylan (BX) and wheat arabinoxylan (WAX) as the sole carbon source. *A. niger* An76 cells grew normally under all carbon sources, with stable growth after 72 h (Additional file 1: Fig. S1). Samples cultured for 0, 6, 12, 24 and 48 h were selected to qRT-PCR analysis, and the results are shown in Fig. 1 and Additional file 1: Fig. S2. Compared with glycerol as the carbon source, when cultured under xylose-like substrate, transcription of three xylanase genes (*xynA, xynB,* and *xynC*) was significantly increased, which indicated their importance to the degradation of xylan.

As shown in Fig. 1a, the relative transcription level of *xynA* was increased significantly at 6 h under xylose, XOS, BX and WAX conditions. Expression of *xynB* was slightly behind that of *xynA,* and the overall transcription level was lower than that of *xynA* (Fig. 1b). In addition, the transcription level of *xynC* was increased at 24 h under xylose, XOS and BX conditions (Fig. 1c). It is worth noting that in WAX, the induction time of *xynC* was earlier, that is, a significant amount of transcription was detected at 12 h, possibly because a higher content of xylan side-chains induced *xynC* expression. Meanwhile, *xynD* and *xynE* were transcribed in small amounts or not at all (Fig. 1d and Additional file 1: Fig. S2). Therefore, xylanases (especially XynA, XynB and XynC) of *A. niger* An76 respond differently to diverse carbon sources and display different secretion orders and substrate preferences. GH11 xylanases degraded xylan backbone into XOS that triggered the subsequent liberation of GH10 xylanase, which hydrolyze the resistant parts of xylan and hydrolysates of the GH11 family [25, 26, 29].

### Sequence analysis of five xylanases secreted by *A. niger* An76

The full-length cDNA of *xynA, xynB, xynC, xynD and xynE* were 585, 621, 906, 636 and 687 bp in length, corresponding to proteins composed of 195, 207, 302, 212 and 229 amino acids, respectively.

**Fig. 2:**
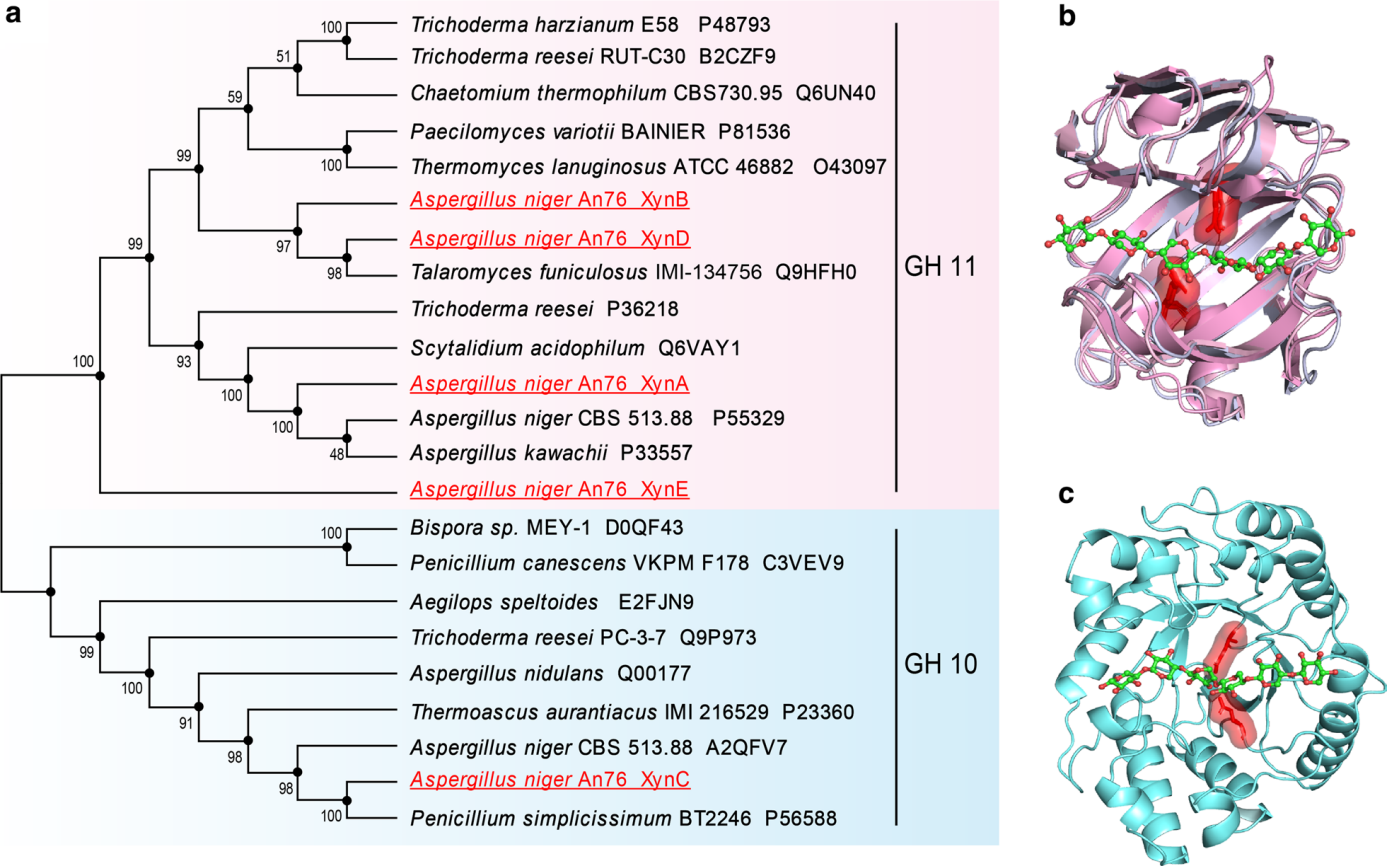
The phylogenetic tree and overall structures of GH11 and GH10 xylanases. **a** Phylogenetic tree resulting from the analysis of amino acid sequences of *A. niger* xylanases and other structurally characterized xylanases from Eukaryota constructed using the neighbor-joining method. Numbers on nodes correspond to the percentage bootstrap values for 1000 replicates. **b** The β-jelly roll structures of XynA, XynB, XynD and XynE from the GH11 family. **c** The (β/α)_8_-barrel structure of XynC from the GH10 family. The substrate sugar ring is represented by a sphere and green sticks. Catalytic amino acids Glu are shown as red sticks

The presence of 16, 18, 19, 19 and 27 amino acid signal peptides at N-terminus were predicted for XynA, XynB, XynC, XynD and XynE, respectively, indicating that these proteins were extracellular enzymes. XynA showed 52.07%, 45.03% and 27.71% amino acid sequence identity to XynB, XynD and XynE, respectively, while XynB showed 65.48% sequence identity to XynD (Additional file 1: Fig. S3). Phylogenetic tree showed that GH10 and GH11 xylanases were divided into two main clades (Fig. 2a). XynA, XynB, XynD and XynE clustered with GH11 family xylanases, and XynC clustered with GH10 family xylanases. In the GH11 family clade, XynB and XynD were located in one subclade, and XynA was located in another subclade. XynB and XynD were located on the same evolutionary branch, indicating that the evolutionary relationship between them is relatively close. But XynE has a relatively distant evolutionary relationship with XynA, XynB and XynD. Xylanases in the GH11 family had a conserved β-jelly roll structure, with a catalytic cleft that could accommodate six xylose moieties, and two key catalytic Glu residues (Fig. 2b). While GH10 xylanases had a conserved (β/α)_8_-barrel structure and these enzymes also had two key catalytic Glu (Fig. 2c).

### Recombinant expression and enzymatic characterization of xylanase isozymes

**Fig. 3:**
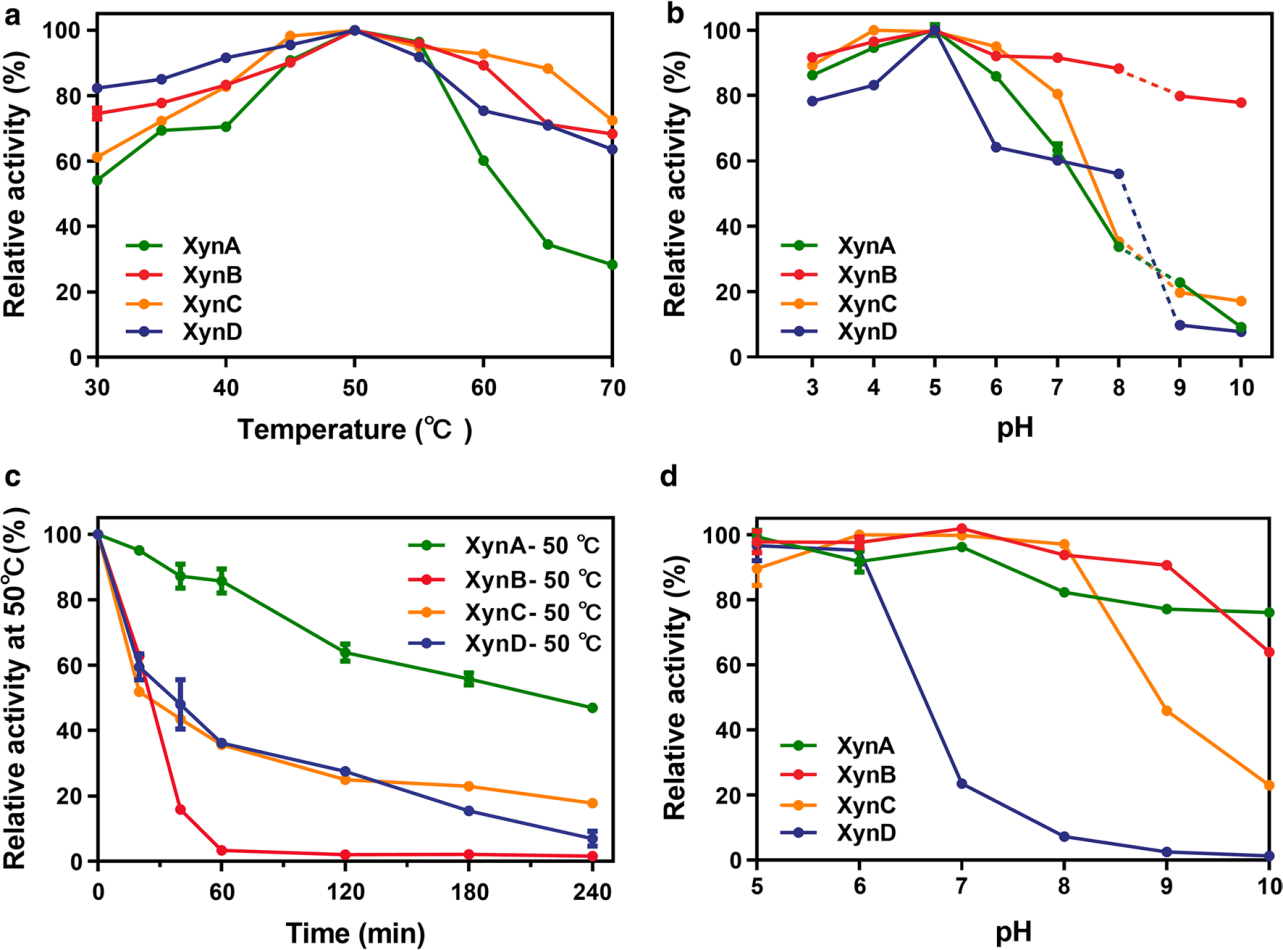
Effect of temperature and pH on the activity and stability of recombinant xylanases. **a** Effect of temperature on the activity of XynA, XynB, XynC and XynD. Enzymes were incubated at 30 °C to 70 °C for 10 min at pH 5.0. **b** Effect of pH on activity of XynA, XynB, XynC and XynD. Enzymes were incubated at pH 3 to 10 for 10 min at 50 °C. **c** Temperature stability of XynA, XynB, XynC and XynD. Residual activities were measured under optimal conditions after incubation at 50 °C for different durations. **d** pH stability of XynA, XynB, XynC and XynD. Residual activities were determined after 30 min incubation at pH 5 to 10 on ice without substrate. Error bars are given as means and standard deviations

To explore the biochemical properties and functions of xylanase isozymes, recombinant enzymes were cloned and expressed using *E. coli* expression system. The purified GH11 xylanases were examined by SDS-PAGE (Additional file 1: Fig. S4). Unfortunately, XynE did not exhibit any xylan-degrading activity, the biochemical analyses were not determined. The optimum temperature and optimum pH of the four xylanases (XynA, XynB, XynC and XynD) were measured using 1% BX as substrate. As shown in Fig. 3a, b, for all enzymes the optimum temperature was 50 °C, and the optimum pH was 5.0. However, they had different tolerances to the same temperature. As shown in Fig. 3c, XynA, XynC and XynD could withstand a temperature of 50 °C, and retained 85%, 35%, and 35% of their respective activities after incubation at 50 °C for 60 min, while XynB has almost lost the activity. Regarding pH stability, XynA and XynB had the best pH tolerance, and were stable at pH 5–10 after incubation for 30 min without substrate (Fig. 3d). XynC was stable at pH 5–8, but their stabilities had decreased in extreme alkaline conditions (pH 9–10). XynD showed the lowest tolerance to alkaline conditions (pH 7–10).

### Substrate specificity and kinetic parameters of xylanase isozymes

To obtain a better understanding of the functions and properties of each GH11 xylanase, the specificity and kinetic parameters were investigated using various substrates. The enzymatic activities of the three xylanases determined using two purified xylan (BX and WAX) and two natural biomass xylan (wheat bran and corn cob) as substrates. As shown in Additional file 1: Fig. S5, XynA, XynB and XynD displayed higher enzymatic activities toward purified xylan and lower enzymatic activity toward natural substrates. Among the three enzymes, XynB showed the highest enzyme activity. The specific activities of XynB for BX (1146.3 IU/mg), WAX (1019.3 IU/mg) are listed in Table 1. The activity of XynB with BX was 6.7-fold higher than that of XynD and 8.2-fold higher than that of XynA. The activity of XynB with WAX was ~ 5.8-fold higher than that of XynD and 10.8-fold higher than that of XynA.

**Table 1:** The kinetic parameters of the three recombinant xylanases

Enzyme	Substrate	Specific activity (IU/mg)	*V*_max_ (μmol/min/mg)	*K*_m_ (mg/mL)	*k*_cat_ (s^−1^)	*k*_cat_/*K*_m_ (mL/mg/s)
XynA	Beechwood xylan	139.9	1446.0	10.7	481.8	45.2
XynB	Beechwood xylan	1146.3	9330.0	3.0	3460.3	1140.9
XynD	Beechwood xylan	170.5	2813.0	5.4	1080.2	199.5
XynA	Wheat arabinoxylan	99.6	904.3	10.2	301.3	29.5
XynB	Wheat arabinoxylan	1019.3	5172.0	3.7	1918.2	512.3
XynD	Wheat arabinoxylan	160.8	1319.0	6.3	506.4	79.9

Kinetic parameters were determined for XynA, XynB and XynD using BX and WAX as substrates (Table 1). The catalytic kinetic parameters of the three GH11 xylanases were quite different. The *K*_m_ value of XynB was the smallest, followed by XynD, and the *K*_m_ value of XynA was the largest. This result indicated that the substrate-binding ability of the three xylanases was ordered: XynB > XynD > XynA. XynB exhibited the largest *k*_cat_, the smallest *K*_m_, and the highest catalytic efficiency (*k*_cat_/*K*_m_), which is consistent with the determination of enzyme activity. The *k*_cat_/*K*_m_ of XynB with BX was 25.3-fold higher than that of XynA and 5.7-fold higher than that of XynD. The *k*_cat_/*K*_m_ of XynB with WAX was 17.3-fold higher than that of XynA and 6.4-fold higher than that of XynD. Moreover, XynA, XynB and XynD all displayed higher catalytic efficiency (*k*_cat_/*K*_m_) for BX than WAX, suggesting that the complexity of substrate structure is inversely proportional to enzyme activity.

### Degradation pattern determination of the three GH11 xylanases

To explore the differences in degradation patterns of the three xylanases, X4, X6, BX, WAX, wheat bran, and corn cob were used as substrates for FACE analysis. Obvious differences in hydrolysis products were observed between xylan substrates (Additional file 1: Fig. S6). The main products of the degradation of BX were xylooligosaccharides with a degree of polymerization (DP) below 5. The hydrolysis products of wheat arabinoxylan contain more large oligosaccharides with DP greater than 5 and less X1, X2, X3. The hydrolysis products generated from wheat bran and corn cob were similar to those WAX, except that the hydrolysate of XynB contained fewer X4 and more X3. These results indicated that xylan containing more side chains inhibited degradation by all three GH11 xylanases, and more importantly, XynB might be advantageous for the degradation of XOS, especially X4.

**Fig. 4:**
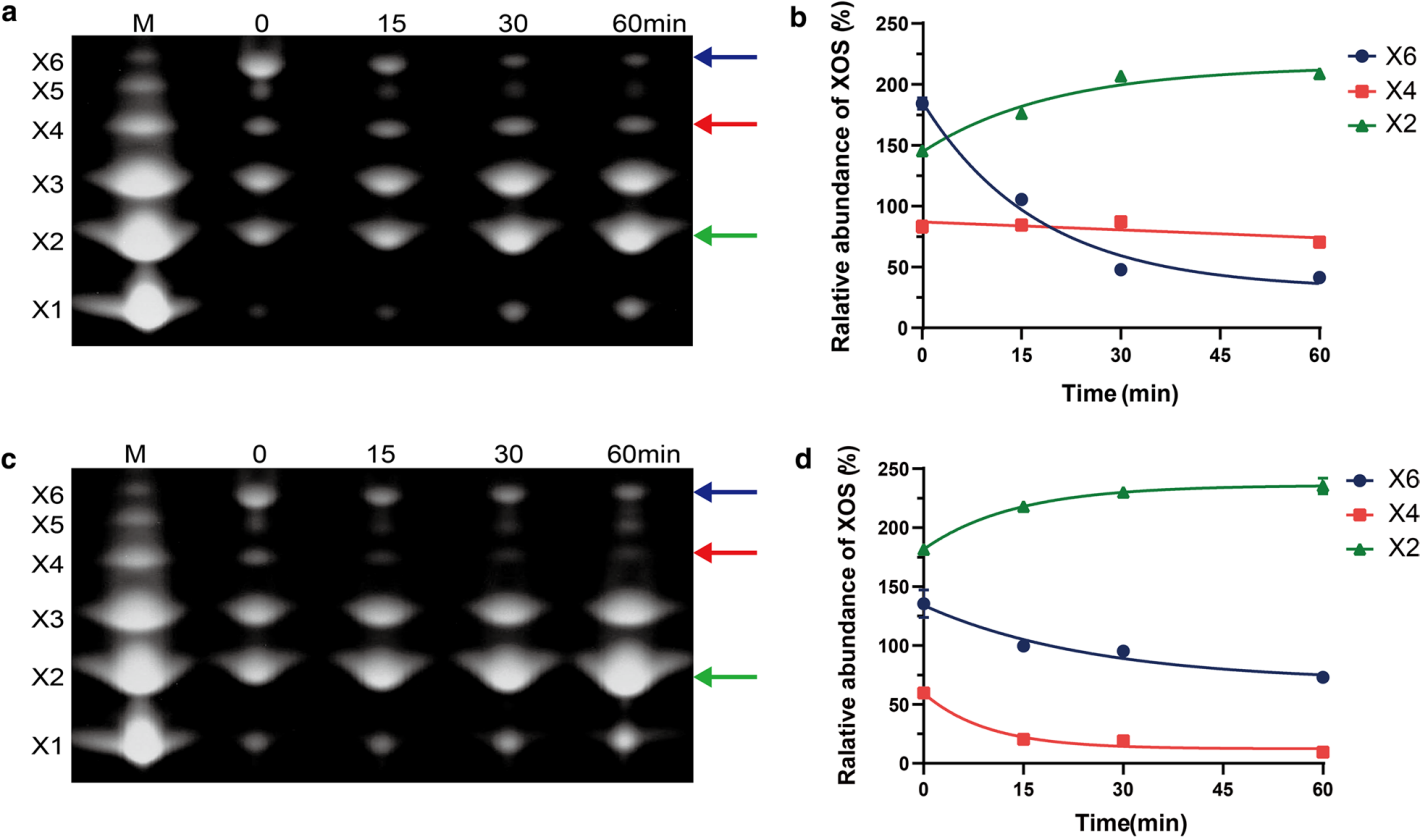
FACE analysis of products following hydrolysis of xylohexaose (**a**, **b**) and xylotetraose (**c**, **d**). **a**, **c** Correspond to the products of xylohexaose hydrolyzed by XynA and XynB, respectively. **b**, **d** Correspond to quantitative analysis of X6, X4 and X2, respectively. Xylose (X1), xylobiose (X2), xylotriose (X3), xylotetraose (X4), xylopentaose (X5), xylohexaose (X6). Blue arrows indicate X6, red arrows indicate X4, and green arrows indicate X2

In the degradation of X4 and X6, XynA degraded X6 faster than XynB and the main products were X2, X3 and X4 (Fig. 4a, b), which indicated that XynA had a preference for X6 degradation. The main products produced from X6 degradation by XynB were X2 and X3 (Fig. 4c, d). From these results, we speculated that XynB may rapidly degrade the produced X4 to generate X2. Analysis of the degradation of X4 confirmed our conjecture. XynA degraded some X4 within 5 min, and the content of X4 remained stable thereafter (Additional file 1: Fig. S7a). In contrast, XynB and XynD continued to degrade X4 over 60 min, and the amount of X2 continued to increase (Additional file 1: Fig. S7b). In addition, the degradation patterns of XynD on X4 and X6 were similar to those of XynB (Additional file 1: Fig. S7c, d).

### Bioinformatic analysis of the active site architecture of GH11 xylanases

**Fig. 5:**
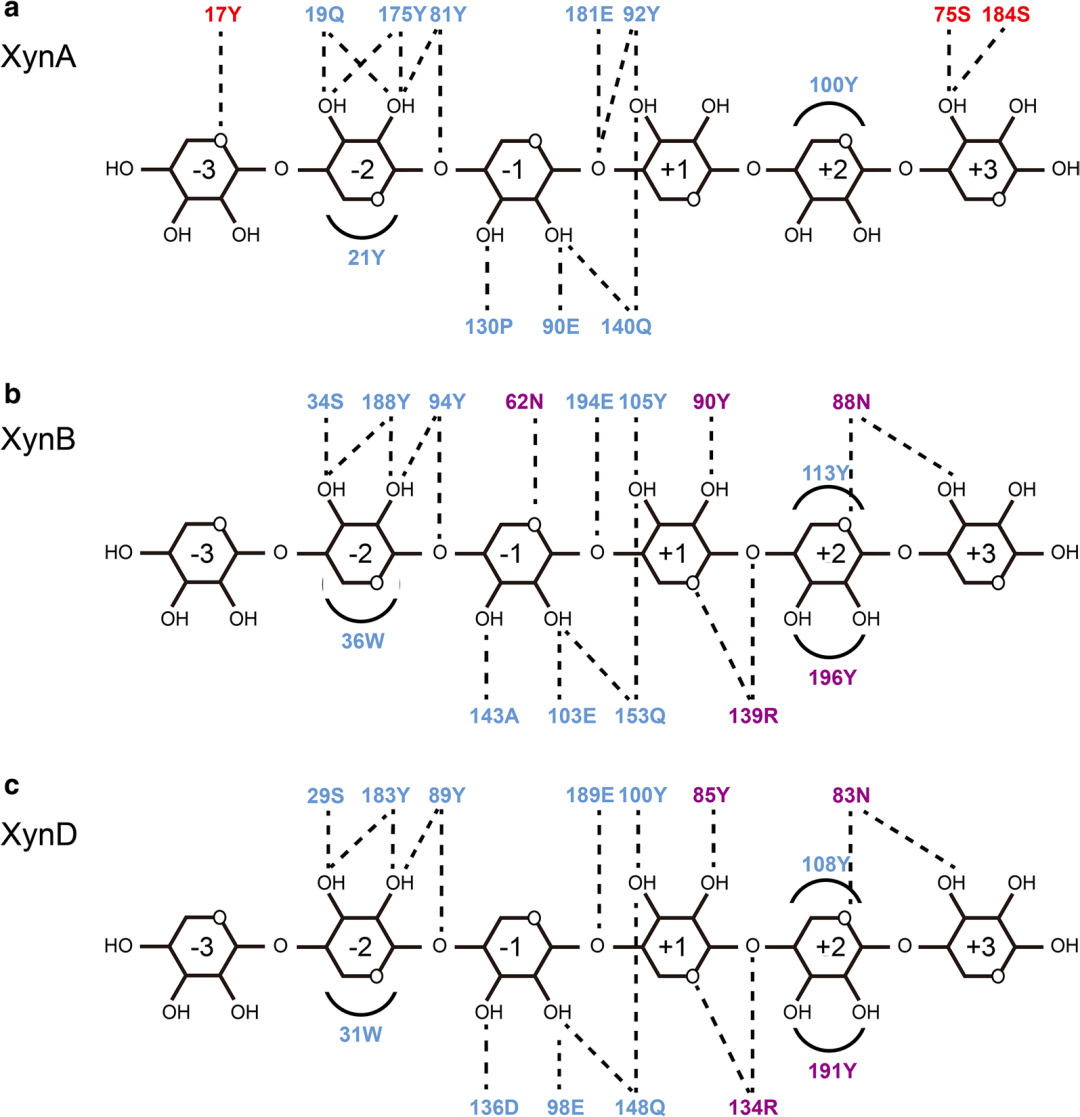
Schematic illustration of interactions between residues in the active site of XynA (**a**), XynB (**b**), XynD (**c**) and the xylohexaoses. Blue amino acids represent common interactions among the three xylanases. Red amino acids represent unique interaction residues of XynA. Purple amino acids represent the unique interaction residues of XynB and XynD. Dashed lines represent polar interactions, and arcs represent CH–π interactions

The catalytic functions of enzymes were closely related to their structures, especially active site architectures. The intricate hydrogen-bonding network formed between key functional amino acids and substrates in the active site architecture of members of the GH11 family was analyzed by structural bioinformatics. As shown in Fig. 5, the active sites of xylanases in GH11 family could accommodate six xylose units, but the number of interactions with the glycosylates at different subsites were different. In the active site architecture of XynA, functional residues 17Tyr at the − 3 subsite and 75Ser, 184Ser at the + 3 subsite contributed to the binding of X6 (Fig. 5a). In contrast, interactions of XynB and XynD were mainly concentrated at the − 2 to + 2 subsites, which contained 4–5 additional interacting amino acids compared with XynA (Fig. 5b, c). These amino acids were 62Asn at the − 1 subsite, 90Tyr, 139Arg at the + 1 subsite, 196Tyr, 88Asn at the + 2 subsite in the active site architecture of XynB (Fig. 5b). It is likely that XynA was dominant in degrading long oligosaccharides and XynB preferred to degrade short oligosaccharides. Altogether, our data showed that the active site architectures of XynA, XynB, and XynD indeed have different interaction networks, which could directly affect the binding capacities and catalytic activities.

### Synergistic hydrolysis of the three GH11 xylanases

**Fig. 6:**
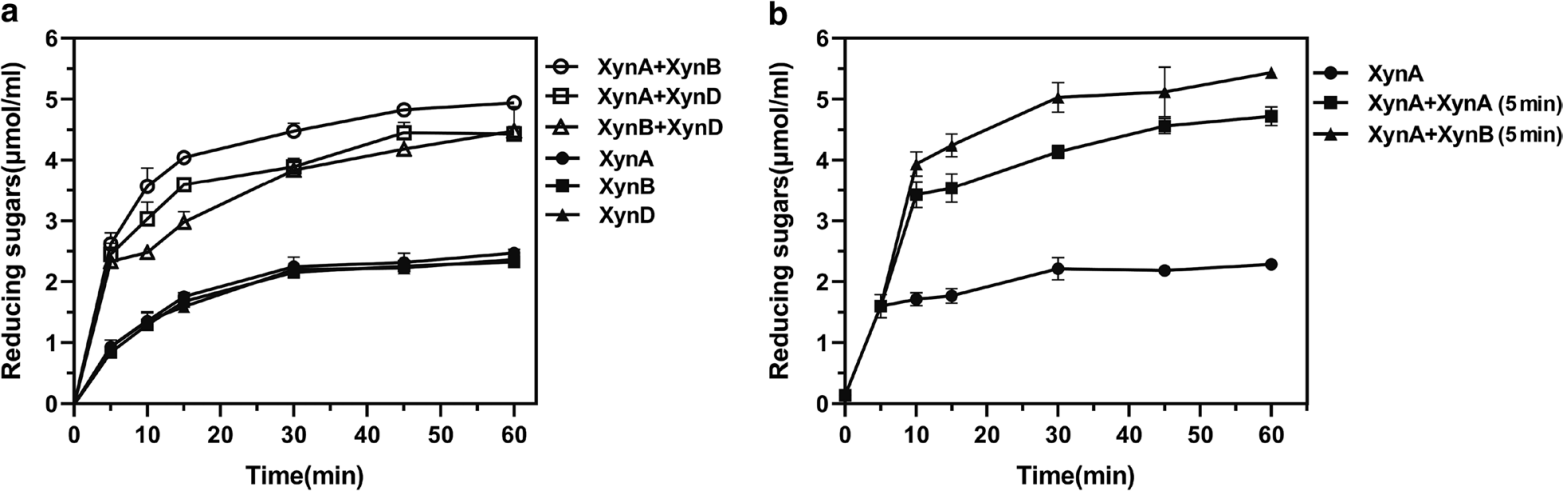
Hydrolysis synergy determination of the three GH11 xylanases. **a** The amount of reducing sugars released when three xylanases were added to BX alone (XynA, XynB, XynD) or in combination (XynA + XynB, XynA + XynD, XynB + XynD). **b** XynA was added into the BX solutions in advance for 5 min, then XynA and XynB were added again into its original solutions with the same enzyme amount to form the groups of XynA + XynA (5 min) and XynA + XynB (5 min). The amount of released reducing sugars were detected at different time points

To further elucidate the functions of the three GH11 xylanase isozymes XynA, XynB and XynD in the degradation of xylan, we conducted synergistic hydrolysis experiments. As shown in Fig. 6a, the degradation efficiency of the pairs of enzymes was higher than that of a single enzyme, and the order of synergistic effect was XynA + XynB > XynA + XynD > XynB + XynD. This result showed that the addition of either XynB or XynD could improve the degradation efficiency of XynA, which might be related to the degradation pattern of XOS. In addition, XynA + XynB (5 min) means that XynA was added to the solution for 5 min and then XynB with equal enzyme activity was added. This treatment resulted in the highest quantity of reducing sugar (Fig. 6b), which indicated that the synergistic hydrolysis efficiency was greatly improved. This result revealed that the sequential secretion of XynA and XynB was a smart strategy for the efficient degradation of xylan by *A. niger* An76.

## Discussion

A variety of microorganisms harbor multiple xylanases with different specific functions that are produced in the presence of lignocellulosic materials for more efficient degradation [12, 26, 30]. Liao et al. [31] demonstrated that the production of multiple xylanases in *Penicillium oxalicum* GZ-2 was attributed to the genetic redundancy of xylanases and the post-translational modifications. Furthermore, the CAZymes of *C. japonicas* that belong to the same GH family are not functionally redundant but have unique physiological functions [32]. In our previous studies, *A. niger* An76 could secret three xylanases (XynA, XynB and XynC) in the presence of xylan substrates [27, 28]. However, there are five xylanase genes in its genome. It has remained unknown how these xylanases contribute to the degradation of xylan. Herein, quantitative transcriptional analysis was used to explore the expression of these five xylanases. *xynA*, *xynB* and *xynC* genes were strongly upregulated under all substrates (Fig. 1), which is consistent with previous proteomic results [27, 28]. The secretion sequence of XynA, XynB and XynC indicated that *A. niger* regulates xylanases expression in an orderly manner, which may be related to the substrate specificities and degradation patterns of xylanases. Previous studies showed that GH10 xylanases can degrade the xylan near crystalline cellulose, which occurred after degradation by GH11 xylanases [25, 32]. This secretion order may contribute to the ability of *A. niger* An76 to degrade xylan substrates more efficiently and synergically. Moreover, xylose could effectively induce xylanase genes transcription by activating the transcription factor XlnR [33]. Analysis of XlnR binding sites in the 1000 bp transcription regulatory region revealed four XlnR binding sites in *xynA* and two XlnR binding sites in *xynB* (Additional file 1: Table S1) [34]. XynA and XynB may be directly dependent on regulation by XlnR and continuously highly expressed. To elucidate the specific transcriptional regulation mechanism, genetic manipulation methods will be needed in future work.

The enzymatic characterization of XynA, XynB, XynC and XynD in the present study showed that the optimum temperature of all xylanases is 50 °C, and the optimum pH of all xylanases is 5.0. In general, fungal xylanases, such as Xyl2 from *Penicillium chrysogenum* P33 [26], Xyn11B from *Penicillium oxalicum* GZ-2 [31] and rXyn162 from *Pleurotus ostreatus* HAUCC 162 [35], have an optimal pH value of about 5.0 and optimal temperature of about 50 or 55 °C. Interestingly, XynE showed no xylanases activity. Combined with the results of previous studies, transcription analysis showed that *xynE* was not induced by xylan substrates (Additional file 1: Fig. S2) and in the phylogenetic tree, XynE is far related to other GH11 xylanases (Fig. 2a). Therefore, we speculate that *xynE* might be a redundant gene. Characterization of XynA, XynB and XynD revealed clear differences in enzyme activities (Additional file 1: Fig. S5 and Table 1). The degree of side-chain substituents of BX and WAX was 12.8% and 38%, and the ratio of xylose to arabinose in natural substrates such as wheat bran was 0.7:1 [28]. The activities of XynA, XynB and XynD with BX were higher than those with WAX, and far higher than those with natural biomass xylan. Therefore, differences in enzyme activity with different substrates may be related to the structural complexity of substrates. These results are similar to those of a previous report on xylanase degradation of different xylan from *Streptomyces* sp. B6 [36]. Moreover, the enzyme activity of XynB (1146.3 IU/mg, 1019.3 IU/mg) was higher than that of XynA (139.9 IU/mg, 99.6 IU/mg) and XynD (170.5 IU/mg, 160.8 IU/mg) in BX and WAX (Table 1). The activity of xylanases derived from some fungi was lower than 1000 IU/mg in most cases [26, 37]. Of course, the thermal stability of XynB needs to be improved through rational design to make it more suitable for degrading pretreated xylan substrates.

Functional differences of xylanases in GH10 and GH11 families have been widely studied [11, 25, 26]. There are few reports on the functional diversity of multiple GH11 xylanases from the same microorganism. The hydrolysis products profiles of GH11 xylanases showed that XynA preferentially degraded X6 to produce X2, X3 and X4, while XynB and XynD preferentially degrade X4 (Fig. 4). Structural bioinformatics analysis revealed the fine distribution of functional residues in active site architectures was diverse (Fig. 5), which might be related to individual degradation pattern. This result correlated with the observed differences in the number of hydrogen bonds between functional amino acids in the active site architecture. The preference of XynA for X6 can be explained by hydrogen bonds at the − 3 and + 3 subsites, consistent with previous studies showing that increasing the binding energy of the enzyme at distal subsites can enhance the ability to degrade long oligosaccharides [38, 39]. In contrast, interactions of XynB and XynD were mainly concentrated in the − 2 to + 2 subsites, which resulted in strong binding of X4 [36]. Different hydrolysis products were generated by different family members [40], and members of the same family can also produce different products due to the low sequence conservation of distal active subsites. Therefore, the fine distribution of functional amino acids in the active sites of isozymes in the same family leads to differences in the number of hydrogen bonds, which may lead to differences in the preference of enzymes for substrates and consequently the diversity of products.

In the hydrolysis of BX substrate, a higher synergistic effect was observed between XynA and XynB (Fig. 6), which was consistent with the different action modes of these two xylanases (Fig. 4). Combined with the results of transcriptional analysis, *xynA* and *xynB*, responded differently to carbon sources, with *xynA* as the pioneer and *xynB* as the second (Fig. 1). The secretion order and functional diversity of xylanase isozymes might be the synergistic mechanism of *A. niger* An76 for the efficient degradation of xylan main-chain. In the present work, we provided a strategy to investigate the functional mechanism underlying the secretions of multiple xylanase isozymes of the GH11 family in *A. niger* An76. First, the substrate preferences and response times of isozymes were analyzed by quantitative transcription. And the biochemical characterization and modes of action were measured experimentally. Functional amino acids and interaction networks with substrates in the active site architecture of isozymes were further analyzed by structural bioinformatics; finally, we performed synergistic hydrolysis of xylanase isozymes. Therefore, the study strategy we developed may help in the selection of suitable enzymes in cocktails.

## Conclusions

This work comprehensively studied synergistic degradation on xylan-backbone by GH11 xylanase isozymes of *A. niger* An76 using quantitative transcriptional analysis and biochemical determination. Our results indicated that the secretion of xylanases may be specifically regulated during the process of biomass degradation. Notably, the features of active site architectures confer different enzymatic activities and modes of action of xylanase isozymes. These findings may help to improve our understanding of the structure–function relationships of xylanases and guide the development of efficient enzymatic cocktails for the degradation biomass.

## Methods

### Strains and growth conditions

*Aspergillus niger* An76 (DDBJ accession no. BCMY00000000, DNA Data Bank of Japan) was used for enzyme gene amplifications. Cultivation of *A. niger* An76 was performed according to methods described previously [27, 41]. Fresh conidia (1 × 10^6^ /mL) were used to inoculate in 250 mL liquid medium in triplicate at 30 °C and 200 rpm. A 1% (w/v) solution of different carbon sources (glycerol, xylose, XOS, BX and WAX) was added to the culture medium to determine the growth of *A. niger* An76 at different time points. For qRT-PCR experiments, washed mycelia, which were cultivated in the presence of 1% (w/v) glycerol as carbon source for 24 h as a reference sample (0 h), were transformed into the fresh media containing 1% (w/v) glycerol, xylose, XOS (DP 2–6, Futian Pharmaceutical Co., Ltd, Shandong, China), BX or WAX (Megzyme, Wicklow, Ireland) for induction. Mycelia samples were collected at different sampling times (0, 6, 12, 24 and 48 h) and stored at − 80 °C.

### RNA isolation and cDNA synthesis

Mycelia sampled at each time point were used to extract total RNA and synthesize cDNA. Total RNA was extracted using TRIzol reagent (Sangon, Shanghai, China) and other regents including chloroform, isopropyl alcohol and ethanol (Dingguo, Beijing, China) using the TRIzol method [42]. The cDNA was synthesized using 1 μg RNA as a template and purified using the HiScript III RT SuperMix + gDNA wiper (Vazyme, Nanjing, China) according to the manufacturer’s instructions. The concentration and purity of total RNA and cDNA were determined by measuring UV absorbance with the Nanophotometer^®^ N60 (Implen, Munich, Germany).

### qRT-PCR analysis of gene expression

qRT-PCR was performed using SYBR qPCR Master Mix (Vazyme) on LightCycler 480 instrument (Roche, Basel, Switzerland). The primers used to detect the expression levels of encoding genes are listed in Additional file 1: Table S2. Target genes included five xylanase genes (*xynA*, g9709.t1; *xynB*, g10033.t1; *xynC*, g1233.t1; *xynD*, g1345.t1; *xynE*, g3744.t1). The glyceraldehyde-3-phosphate dehydrogenase gene (*gapdh*, g7576.t1) was used as an internal reference gene. Error bars indicated the standard deviation. The concentration of the cDNA template was 20 ng/μL. Relative transcription levels of target genes were calculated by the relative quantitation (2^−ΔΔCT^) method [43]. Three biological replicates and technical replicates were performed.

### Gene cloning, enzyme expression and purification

Xylanase genes were cloned using PCR with synthesized cDNA as the template and primers listed in Additional file 1: Table S2. PCR products were purified and cloned into a pLYJ-163 vector (*xynA*, *xynB*, xynC, *xynD*) and pEASY-Blunt E1 vector (*xynE*) to construct recombinant plasmids, which were transformed into *E. coli* DH5α cells and confirmed by DNA sequencing (TSINGKE, Qingdao, China). The correct recombinant plasmids were transformed into *E. coli* BL21 (DE3) for protein expression. When the absorbance at 600 nm (OD_600_) reached 0.6–0.8, strains were induced using 0.5 mM isopropyl-β-d-thiogalactopyranoside (IPTG, Solarbio, Beijing, China) for 20 h at 16 °C. Cells were centrifuged and resuspended in buffer (50 mM NaH_2_PO_4_, 300 mM NaCl, pH 8.0). After ultrasonic fragmentation, xylanases were purified using HisCap Co 6FF resin (Smart-life Sciences, Changzhou, China). The eluent was replaced with optimal buffer (50 mM Na_2_HPO_4_, 20 mM citrate, pH 5.0) by ultrafiltration (3 kDa membrane, Millipore, Billerica, MA) at 4 °C. Pure enzymes were analyzed by sodium dodecyl sulfate polyacrylamide gel electrophoresis (SDS-PAGE). Protein concentrations were determined using the Bradford method [44].

### Enzymatic activity assay

Xylanase activity was assayed using the 3,5-dinitrosalicylic acid (DNSA) method [45]. A 40 μL volume of appropriately diluted XynA, XynB, XynC and XynD (5 μg/mL, 1 μg/mL, 5 μg/mL and 5 μg/mL, respectively) were incubated with 60 μL 1% (w/v) xylan substrates. After incubation at 50 °C for 10 min, samples were mixed with 80 μL of DNSA reagent, boiled immediately for 10 min, and subsequently cooled on ice. After adding 820 μL water, the absorbance were measured at 540 nm. One unit of xylanase activity (IU) was defined as the amount of enzyme (mg) that released 1 μmol of xylose per minute under the optimal assay conditions.

### Effects of pH and temperature on xylanases

The optimal pH of xylanases was determined in the range of pH 3.0–10.0 (50 mM sodium citrate buffer, pH 3.0–8.0; 50 mM Glycine–NaOH buffer, pH 9.0–10.0) by adding BX substrate and incubating for 10 min at 50 °C using the above activity assay method. The activity at the optimal pH was defined as 100%. The optimal temperature of xylanases was determined at temperatures ranging from 30 to 70 °C in sodium citrate buffer (pH 5.0). The activity at the optimal temperature was defined as 100%.

The temperature stability of xylanases was determined by measuring the remaining activity after incubating enzymes for various times durations (0, 20, 40, 60, 120, 180 and 240 min) at 50 °C in the absence of BX in 50 mM sodium citrate buffer (pH 5.0). The activity without pre-incubation was defined as 100%. To assess pH stability, xylanases were incubated in pH 5.0–10.0 buffers for 30 min, and the remaining enzyme activities were determined under optimal conditions. All experiments were performed in triplicate.

### Substrate specificity and determination of kinetic parameters

Substrate specificity of xylanases was investigated using different xylan substrates at 1% concentration, including BX, WAX, xylan from wheat bran and corn cob extracted by alkaline pretreatment [46]. Solid material was washed, 0.7% NaOH was added at a ratio of 1:7, and samples were incubated at 60 °C for 2 h. The resulting mixtures were filtered and the filtrate was neutralized with HCl. Xylan was precipitated with absolute ethanol, dried, ground into a powder, and stored at room temperature for subsequent experiments. Reactions were performed in 50 mM sodium citrate buffer (pH 5.0) at 50 °C for 10 min.

The kinetic parameters (*K*_m_ and *V*_max_) of the enzymes were determined by incubating with different concentrations of BX and WAX (0.2–2.4%) at 50 °C for 5 min. Parameters were calculated using nonlinear regression of the Michaelis–Menten equation with GraphPad Prism 8.0 [47]. All experiments were performed in triplicate.

### Analysis of hydrolysis products.

Hydrolysis products of different xylan substrates were determined by fluorescence-assisted carbohydrate electrophoresis (FACE) experiments [48]. Hydrolysis products were obtained following the reaction of enzymes (20 IU/mL) with 1% substrates at a ratio of 1:1 (v/v) at 50 °C. Samples were removed at different time points (0, 5, 15, 30 and 60 min) and inactivated at 105 °C for 10 min to terminate the reaction. The supernatant was collected by centrifugation at 12,000 rpm for subsequent electrophoresis analysis. Xylose (X1), xylobiose (X2), xylotriose (X3), xylotetraose (X4), xylopentaose (X5), xylohexaose (X6) were used as standards. Band intensity was quantified from peaks of TIF images using Quantity One software (Bio-Rad Laboratories, Hercules, CA).

### Synergistic hydrolysis experiments

To investigate the synergistic effect among xylanases, for each xylanase, the amount of the enzyme with equal xylanase activity (0.5 IU/mL) towards BX was used in experiments. Xylanases were added singly (XynA, XynB, XynD) or in pairs (XynA + XynB, XynA + XynD, XynB + XynD) to 1% BX solution. The amount of reducing sugars released was determined by the DNSA method after incubation at 50 °C for 60 min. Samples were removed at intervals, and samples without enzyme as controls.

Another method was used to determine synergy further between XynA and XynB [25]. XynA was added in advance into the BX substrate and incubated at 50 °C for 5 min, then XynA and XynB with equal xylanase activity were added into the solutions to form the groups named by XynA + XynB (5 min) and XynA + XynA (5 min). The reducing sugars released at different time points were detected during incubation for 60 min. Samples without enzyme under the same conditions served as controls. All the above hydrolysis assays were performed in triplicate.

### Bioinformatics analysis

All characterized xylanases with PDB ID from eukaryota in the CAZy database (http://www.cazy.org/**)** were selected to construct a phylogenetic tree to improve the accuracy of subsequent structure prediction. Signal peptides were predicted using SignalP website (http://www.cbs.dtu.dk/services/SignalP/). Multiple sequence alignments were performed using CLUSTAL (http://www.clustal.org/). Phylogenetic analysis was performed using MEGA 5.0 with the Neighbor-Joining method and 1000 bootstrap replicates [49]. Homology modeling was carried out using SWISS-MODEL (https://swissmodel.expasy.org/). Suitable templates were identified, *Aspergillus niger* CBS 513.88 (PDB ID: 2QZ2), *Talaromyces funiculosus* IMI-134756 (PDB ID: 1TE1), *Aspergillus niger* CBS 513.88 (PDB ID:4XUY), *Talaromyces cellulolyticus* CF-2612 (PDB ID: 3WP3) and uncultured bacterium (PDB ID:2VGD) were selected for XynA, XynB, XynC, XynD and XynE, respectively. Models were then generated based on each of the individual templates. The ligand in the enzyme-ligand complex of GH11 and GH10 was obtained from *Tr*Xyn11A (PDB: 4HK8) and xylanase T6 (PDB: 4PRW), respectively. Amino acid residues surrounding the substrate within 5 Å were selected to analyze the interaction network between enzymes and substrates. The chemical structure diagram was described by ChemDraw (http://www.chemdraw.com.cn/).

## Supplementary Information

**Additional file 1**: **Fig. S1.** Growth condition of *A. niger* An76 on different carbon sources. **Fig. S2.** Gene relative transcript levels of *xynE* induced by 1% glycerol, xylose, XOS, BX and WAX at 0 h, 6 h, 12 h, 24 h and 48 h. **Fig. S3.** Sequence alignment of XynA, XynB, XynC, XynD and XynE with related GH11 and GH10 xylanases. a Alignment of XynA, XynB, XynD, XynE from* A. niger *An76 and XylA from *Aspergillus niger* CBS513.88, XynC from *Talaromyces funiculosus *IMI-134756 and Xyn2 from *Trichoderma reesei* RUT-C30. b GH10 xylanases alignment includes XynC from *A. niger* An76 and XynA from *Aspergillus niger* CBS513.88 and Xyn2 from* Penicillium canescens *VKPM F178. Strictly conserved residues are highlighted by a red background, and conservatively substituted residues are boxed. **Fig. S4.** SDS-PAGE analysis of four GH11 xylanases from *A. niger *An76 expressed in *E. coli *strain BL21 (DE3). Lane M: protein molecular weight marker. **Fig. S5.** The catalytic activities of the three GH11 xylanases on various substrates (beechwood xylan, wheat arabinoxylan, wheat bran and corncob). **Fig. S6**. FACE electrophoresis analysis of the products after hydrolysis of various xylans. Four substrates (beechwood xylan, wheat arabinoxylan, wheat bran, and corn cob) were hydrolyzed by XynA, XynB and XynD for 12 h under optimal conditions. Mixture of xylose (X1), xylobiose (X2), xylotriose (X3), xylotertraose (X4), xylopentaose (X5), xylohexaose (X6) was used as standards makers. **Fig. S7.** FACE analysis of products following hydrolysis of xylohexaose and xylotetraose by XynA, XynB and XynD. a The products of xylotetraose hydrolyzed by XynA. b The products of xylotetraose hydrolyzed by XynB. c The products of xylohexaose hydrolyzed by XynD. d The products of xylotetraose hydrolyzed by XynD. Xylose (X1), xylobiose (X2), xylotriose (X3), xylotetraose (X4), xylopentaose (X5), xylohexaose (X6) was used as standards makers. **Table S1.** Endo-β-1,4-xylanase of *Aspergillus niger* An76. **Table S2.** Primers used in this study.

## Data Availability

The data generated or analysed during this study are included in this published article and its additional information files. Further datasets used and analysed during the current study are available from the corresponding author on reasonable request.

## Abbreviations

GH: Glycoside hydrolase
CBM: Carbohydrate-binding module
XOS: Xylooligosaccharide
BX: Beechwood xylan
WAX: Wheat arabinoxylan
DP: Degree of polymerization
qRT-PCR: Quantitative real-time PCR
FACE: Fluorescence-assisted carbohydrate electrophoresis
X6: Xylohexaose
X5: Xylopentaose
X4: Xylotetraose
X3: Xylotriose
X2: Xylobiose
X1: Xylose
