# Supplementary material for: Synergistic mechanism of GH11 xylanases with different action modes from Aspergillus niger An76
Source: Biotechnol Biofuels. 2021 May 10;14:118. doi: 10.1186/s13068-021-01967-1 (PMC8112042; doi:10.1186/s13068-021-01967-1)
Supplement: Supplementary file 1 — Additional file 1: Fig. S1. Growth condition of A. niger An76 on different carbon sources. Fig. S2. Gene relative transcript levels of xynE induced by 1% glycerol, xylose, XOS, BX and WAX at 0 h, 6 h, 12 h, 24 h and 48 h. Fig. S3. Sequence alignment of XynA, XynB, XynC, XynD and XynE with related GH11 and GH10 xylanases. a Alignment of XynA, XynB, XynD, XynE from A. niger An76 and XylA from Aspergillus niger CBS513.88, XynC from Talaromyces funiculosus IMI-134756 and Xyn2 from Trichoderma reesei RUT-C30. b GH10 xylanases alignment includes XynC from A. niger An76 and XynA from Aspergillus niger CBS513.88 and Xyn2 from Penicillium canescens VKPM F178. Strictly conserved residues are highlighted by a red background, and conservatively substituted residues are boxed. Fig. S4. SDS-PAGE analysis of four GH11 xylanases from A. niger An76 expressed in E. coli strain BL21 (DE3). Lane M: protein molecular weight marker. Fig. S5. The catalytic activities of the three GH11 xylanases on various substrates (beechwood xylan, wheat arabinoxylan, wheat bran and corncob). Fig. S6. FACE electrophoresis analysis of the products after hydrolysis of various xylans. Four substrates (beechwood xylan, wheat arabinoxylan, wheat bran, and corn cob) were hydrolyzed by XynA, XynB and XynD for 12 h under optimal conditions. Mixture of xylose (X1), xylobiose (X2), xylotriose (X3), xylotertraose (X4), xylopentaose (X5), xylohexaose (X6) was used as standards makers. Fig. S7. FACE analysis of products following hydrolysis of xylohexaose and xylotetraose by XynA, XynB and XynD. a The products of xylotetraose hydrolyzed by XynA. b The products of xylotetraose hydrolyzed by XynB. c The products of xylohexaose hydrolyzed by XynD. d The products of xylotetraose hydrolyzed by XynD. Xylose (X1), xylobiose (X2), xylotriose (X3), xylotetraose (X4), xylopentaose (X5), xylohexaose (X6) was used as standards makers. Table S1. Endo-β-1,4-xylanase of Aspergillus niger An76. Table S2. Primers used in this study. [file 13068_2021_1967_MOESM1_ESM.docx]

**Additional files**

**Synergistic mechanism of GH11 xylanases with different action modes from *Aspergillus niger* An76**

Shu Zhang^1^, Sha Zhao^1,3^, Weihao Shang^1^, Zijuan Yan^3^, Xiuyun Wu^1,2^*, Yingjie Li^1^, Guanjun Chen^1^, Xinli Liu^2^, Lushan Wang^1^

^1^State Key Laboratory of Microbial Technology, Institute of Microbial Technology, Shandong University, Qingdao, Shandong, 266237, China

^2^State Key Laboratory of Biobased Material and Green Papermaking，Qilu University of Technology，Shandong Academy of Sciences, Jinan, Shandong, 250353, China

^3^School of Life Sciences, Shandong University, Qingdao, Shandong, 266237, China

***Corresponding author:** Xiuyun Wu

**E-mail:** wuxiuyun3353@163.com

**Fax:** +86**-0**532**-**58631569

**Email addresses:**

Shu Zhang: zhshu177@163.com

Sha Zhao: 1374117784@qq.com

Weihao Shang: 201932447@mail.sdu.edu.cn

Zijuan Yan: 15171148456@163.com

Yingjie Li: sdliyingjie@163.com

Guanjun Chen: guanjun@sdu.edu.cn

Xinli Liu: liuxl@qlu.edu.cn

Lushan Wang: lswang@sdu.edu.cn


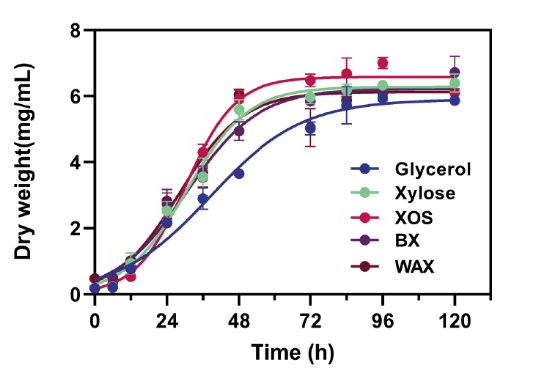


**Fig. S1 Growth condition of *A. niger* An76 on different carbon sources.**

**
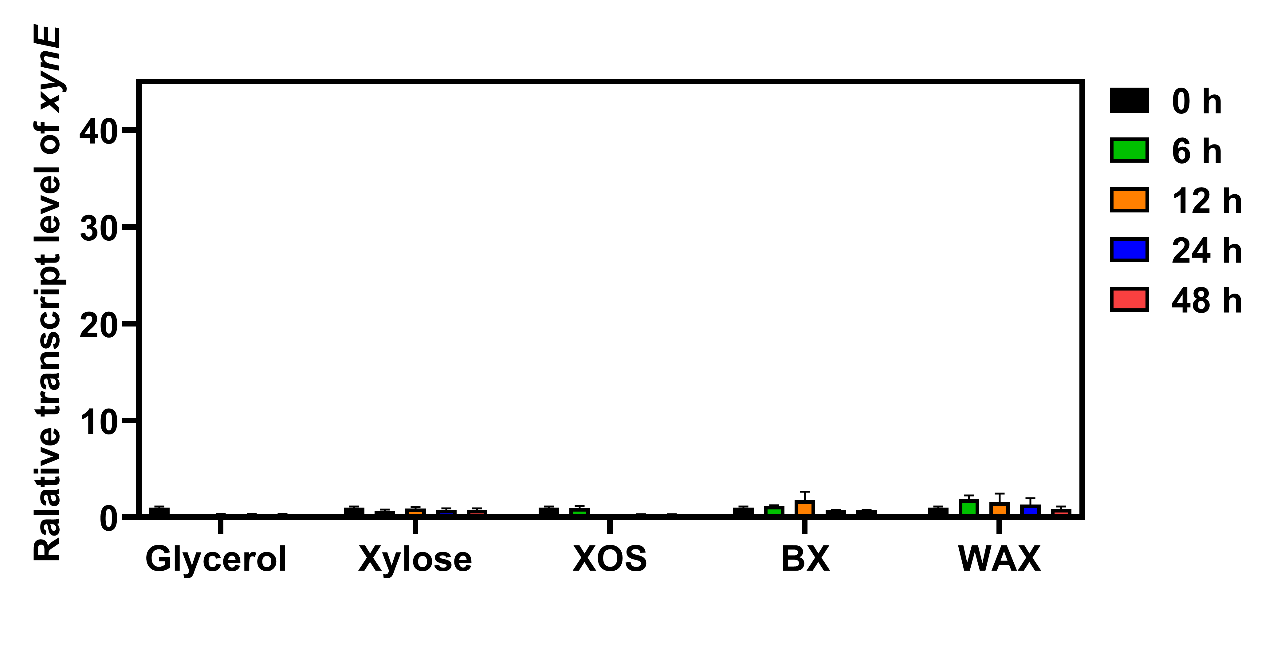
**

**Fig. S2 Gene relative transcript levels of *xynE* induced by 1% glycerol, xylose, XOS, BX and WAX at 0 h, 6 h, 12 h, 24 h and 48 h.**


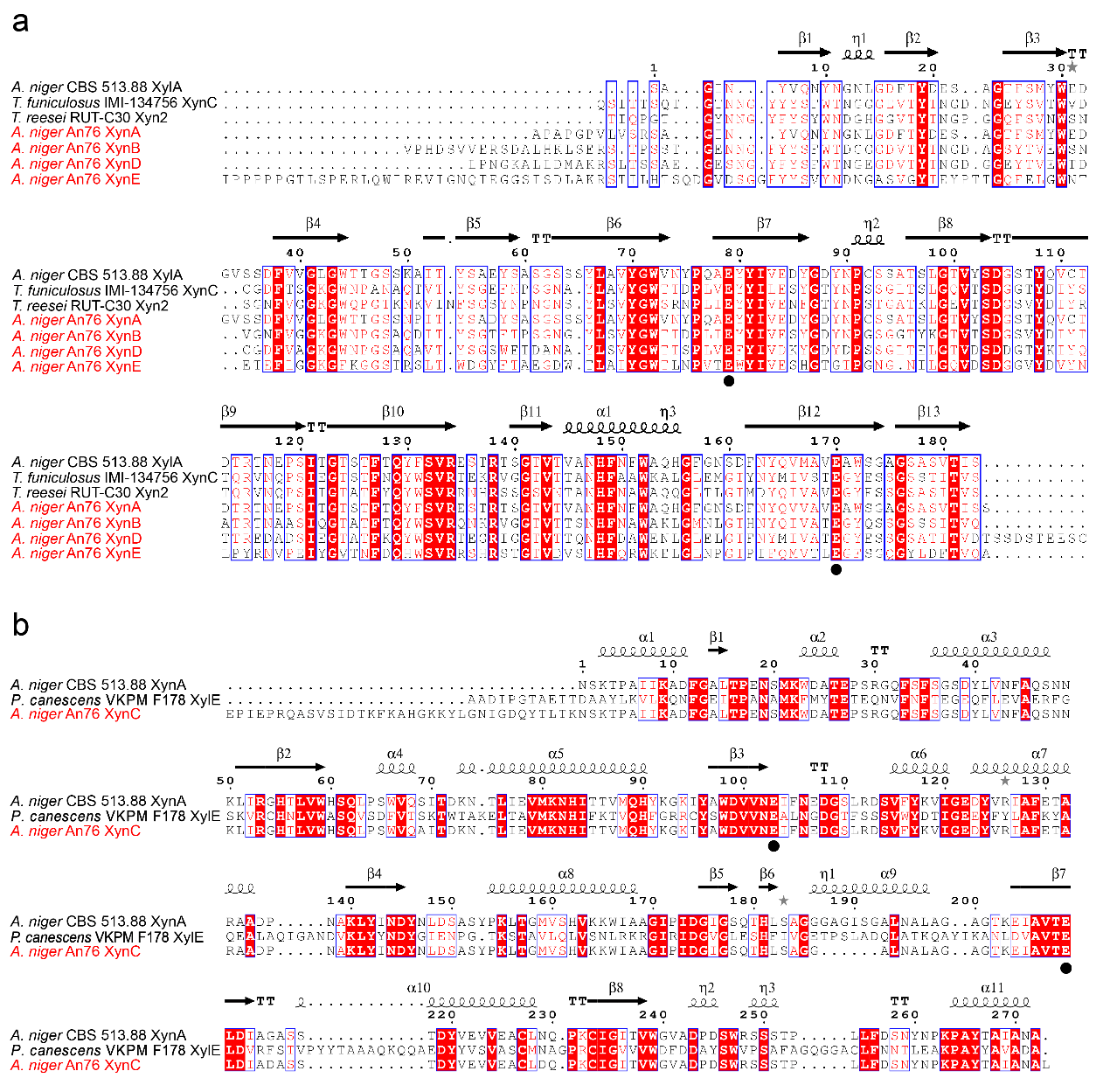


**Fig. S3 Sequence alignment of XynA, XynB, XynC, XynD and XynE with related GH11 and GH10 xylanases. a** Alignment of XynA, XynB, XynD, XynE from *A. niger* An76 and XylA from *Aspergillus niger* CBS513.88, XynC from *Talaromyces funiculosus* IMI-134756 and Xyn2 from *Trichoderma reesei* RUT-C30. **b** GH10 xylanases alignment includes XynC from *A. niger* An76 and XynA from *Aspergillus niger* CBS513.88 and Xyn2 from *Penicillium canescens* VKPM F178. Strictly conserved residues are highlighted by a red background, and conservatively substituted residues are boxed.


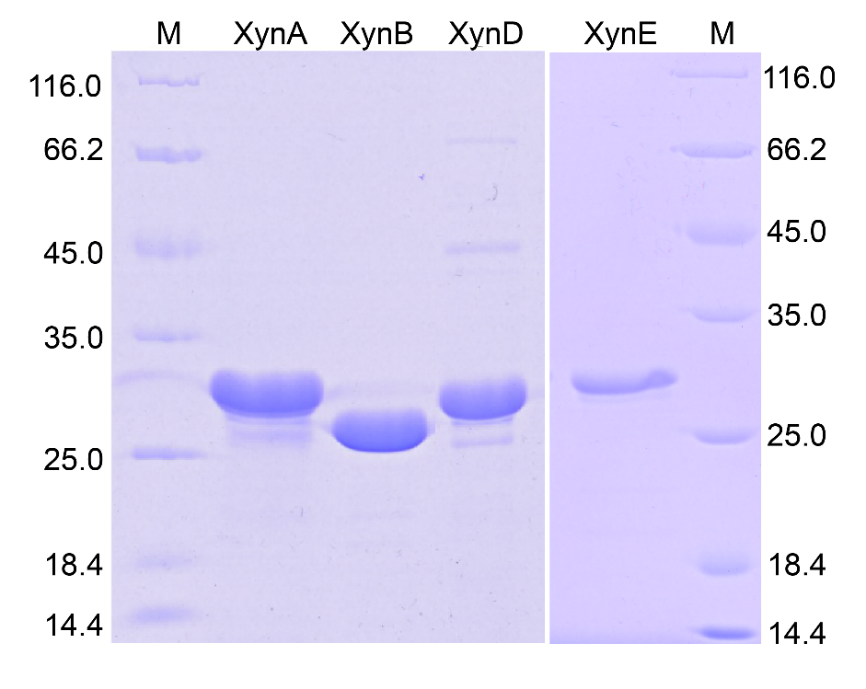


**Fig. S4 SDS-PAGE analysis of four GH11 xylanases from *A. niger* An76 expressed in *E. coli* strain BL21 (DE3).** Lane M: protein molecular weight marker.


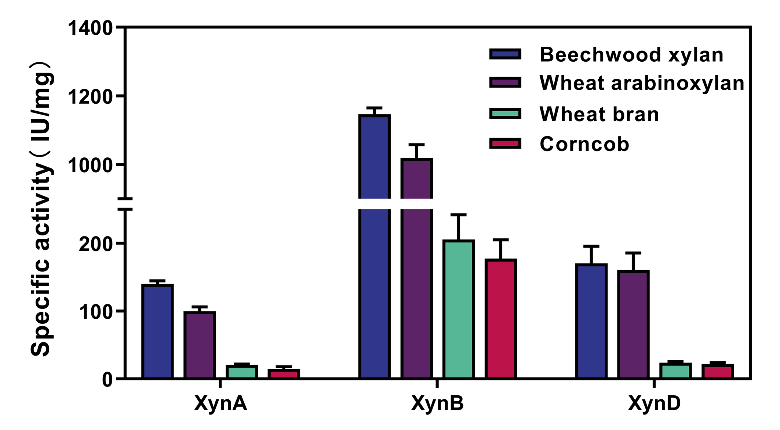


**Fig. S5 The catalytic activities of the three GH11 xylanases on various substrates (beechwood xylan, wheat arabinoxylan, wheat bran and corncob).**


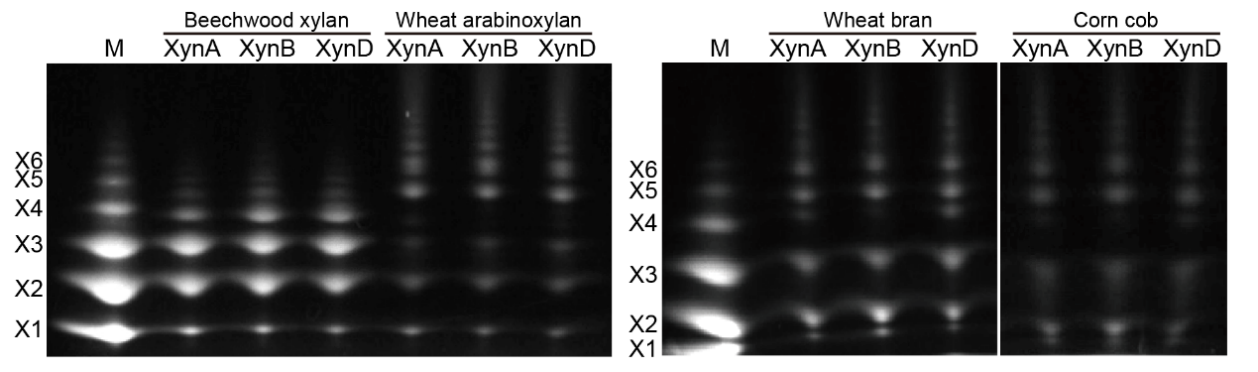


**Fig. S6 FACE electrophoresis analysis of the products after hydrolysis of various xylans.** Four substrates (beechwood xylan, wheat arabinoxylan, wheat bran, and corn cob) were hydrolyzed by XynA, XynB and XynD for 12 h under optimal conditions. Mixture of xylose (X1), xylobiose (X2), xylotriose (X3), xylotertraose (X4), xylopentaose (X5), xylohexaose (X6) was used as standards makers.


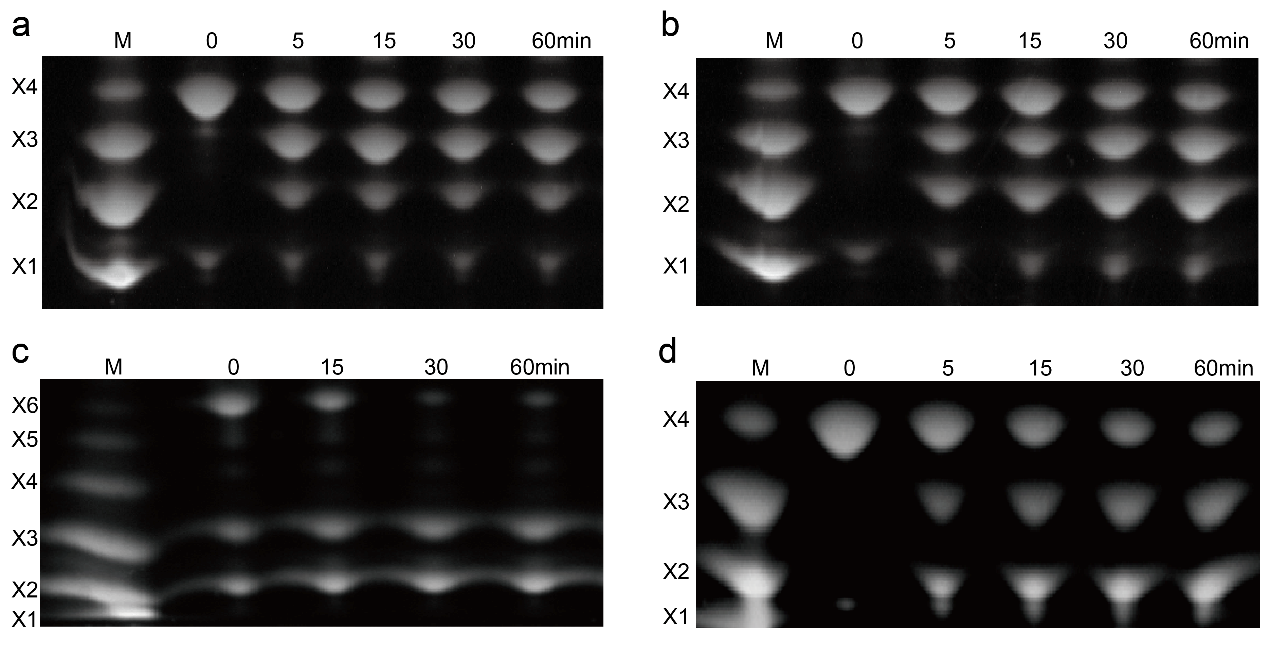


**Fig. S7 FACE analysis of products following hydrolysis of xylohexaose and xylotetraose by XynA, XynB and XynD. a** The products of xylotetraose hydrolyzed by XynA. **b** The products of xylotetraose hydrolyzed by XynB. **c** The products of xylohexaose hydrolyzed by XynD. **d** The products of xylotetraose hydrolyzed by XynD. Xylose (X1), xylobiose (X2), xylotriose (X3), xylotetraose (X4), xylopentaose (X5), xylohexaose (X6) was used as standards makers.

**Table S1 Endo-β-1,4-xylanase of *Aspergillus niger* An76**

| **Gene ID** | **Gene name** | **cDNA*/bp** | **CAZy family** | **EC** | **Signal peptide** | **Number of XlnR binding sites^a^** |
| --- | --- | --- | --- | --- | --- | --- |
| g9709.t1 | *xynA* | 585 | GH11 | 3.2.1.8 | + | 4 |
| g10033.t1 | *xynB* | 621 | GH11 | 3.2.1.8 | + | 2 |
| g1233.t1 | *xynC* | 906 | GH10 | 3.2.1.8 | + | 0 |
| g1345.t1 | *xynD* | 636 | GH11 | 3.2.1.8 | + | 0 |
| g3744.t1 | *xynE* | 687 | GH11 | 3.2.1.8 | + | 0 |

**^a^** Consensus region of XlnR binding sites: 5`-GGCTAATAA or 5`-GGCTAR

**Table S2 Primers used in this study.**

| **Primer** | **Primer purpose** | **Sequence (from 5′ to 3′)** |
| --- | --- | --- |
| xynA-f | Expression primers | CATG*CCATGG*CGGCTCCTGCCCCGGGACCTGTTCTG |
| xynA-r |  | CCG*CTCGAG*TTAAGAAGAGATCGTGACACTGGCGCT |
| xynB-f |  | CATG*CCATGG*CGGTTCCCCACGACTCTGTCGTCGAG |
| xynB-r |  | CCG*CTCGAG*TTACTGAACAGTGATGGAGGAAGATCC |
| xynC-f |  | CATG*CCATGG*GAACCTATTGAACCCCGTCA |
| xynC-r |  | CCG*CTCGAG*TTAGAGAGCATTTGCGATAG |
| xynD-f |  | CATG*CCATGG*CGCTCCCCAACGGTAAGGCCCTGCTG |
| xynD-r |  | CCG*CTCGAG*TTAGCAGCTCTCCTCAGTGCTGTCAGA |
| xynE-f |  | ATCCCCCCACCGCCGCCTGGAACTC |
| xynE-r |  | TTACGCCTGCACGGTGAAATCCAGG |
| gapdh-qf | Quantitative-PCR primers | ATTTTGGTGTTGCTCAGGG |
| gapdh-qr |  | CGGCGGTTCTTCTTGCTAT |
| xynA-qf |  | AAACGAACCGTCCATCACA |
| xynA-qr |  | GCAACAGTCACCGTTCCAG |
| xynB-qf |  | ACGGCTTCTACTACTCCTTCTG |
| xynB-qr |  | AGCCCTTTCCACCAACAA |
| xynC-qf |  | TCGGGATCGGATTACCTG |
| xynC-qr |  | TTTGTCCGTGATGGCTTG |
| xynD-qf |  | ACAGCGGATCTTGGGAAAC |
| xynD-qr |  | CAGAGGAGGGGTCGTAGTCA |
| xynE-qf |  | GACCTGGGACGGTTACTTCA |
| xynE-qr |  | GCCATGCGACTCCACAATA |

The italic sequences indicated the recognition sequence of restriction endonuclease *Nco*I and *Xho*I, respectively.
